# Case report: Potential role of immunotherapy in thymic malignancies: a unique case of a durable and complete response upon an immune checkpoint inhibitor

**DOI:** 10.3389/fimmu.2024.1423800

**Published:** 2024-07-03

**Authors:** Angelo Luciano, Erica Pietroluongo, Margaret Ottaviano, Angela Grieco, Annarita Peddio, Pietro De Placido, Alberto Servetto, Massimo Mascolo, Silvia Varricchio, Roberto Bianco, Giovannella Palmieri, Mario Giuliano

**Affiliations:** ^1^ Department of Clinical Medicine and Surgery, University of Naples Federico II, Naples, Italy; ^2^ Unit of Melanoma, Cancer Immunotherapy and Development Therapeutics, Istituto Nazionale Tumori Istituto di Ricovero e Cura a Carattere Scientifico (IRCCS) Fondazione G. Pascale, Naples, Italy; ^3^ Department of Medical Oncology, Dana-Farber Cancer Institute, Boston, MA, United States; ^4^ Department of Advanced Biomedical Sciences, Unit of Pathology, University of Naples Federico II, Naples, Italy; ^5^ Regional Coordinating Center for Rare Tumors (CRCTR) of Campania Region at University Federico II, Naples, Italy

**Keywords:** immune-related adverse events, autoimmunity, Good’s syndrome, thymomas, immunotherapy

## Abstract

Thymic epithelial tumors are rare malignancies with an incidence of 1.7 cases per million people per year. They pose significant management challenges due to their association with autoimmune disorders. In this case report, we present the 21-year history of a patient diagnosed with advanced B2/B3 thymoma and Good’s syndrome. The patient achieved a complete and durable response after receiving only two cycles of the immune checkpoint inhibitor Nivolumab. However, this positive outcome was accompanied by the development of severe immune-related myocarditis complicated by reactivation of cytomegalovirus. Moreover, the patient developed a highly uncommon subdiaphragmatic pararectal dissemination of the thymic tumor, which is a condition rarely described in the literature. Despite the success in achieving complete and durable response with immune checkpoint inhibitors, the emergence of immune-related adverse events highlights the potential challenges associated with these treatments, emphasizing the need for careful monitoring and a comprehensive understanding of the intricate interplay between cancer, immune system dysregulations and immunotherapy.

## Introduction

Thymic epithelial tumors (TETs) are rare malignancies with an incidence of 1.7 cases per million annually. However, they represent the most prevalent tumors found in the anterior mediastinum among adults (1).

The 5^th^ World Health Organization (WHO) histopathological classification of TETs includes thymomas, thymic carcinomas (TCs) and thymic neuroendocrine tumors (2). Surgery is curative in the early stages, but a combined multimodal approach comprising chemotherapy, radiotherapy and surgery should be considered for advanced disease (3). TETs are a unique model of tumors that arise in a lymphoid organ and involve the entire immune system. Dysfunction of the immune system can manifest at any stage in the history of thymic tumors. Consequently, predicting patient prognosis based solely on neoplastic characteristics is challenging, as it does not account for the variable impact of immune system alterations.

Several autoimmune disorders can be associated with TETs, such as Good’s syndrome (GS). GS is a rare secondary immunodeficiency syndrome characterized by hypogammaglobulinemia, low or absent B cells, reversal of the CD4+/CD8+ T-cell ratio and an impaired T-cell-mediated response with increased susceptibility to bacterial, viral and fungal infections (4, 5).

Here, we present a case of a patient with advanced B2/B3 thymoma and GS who achieved a durable and complete response with an immune checkpoint inhibitor (ICI) after several prior lines of platinum-based chemotherapy.

## Case presentation

**Figure 1:**
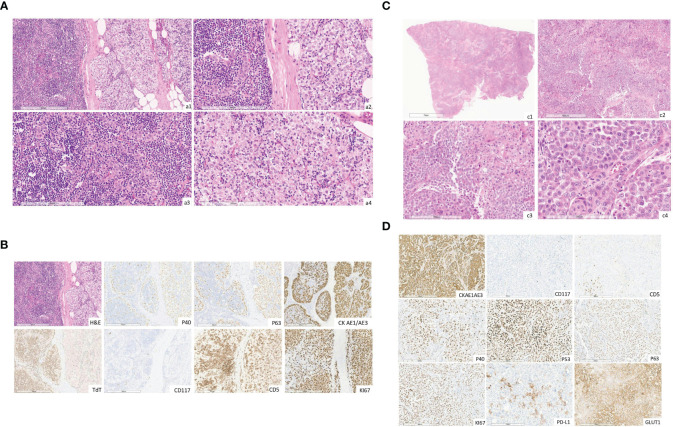
**(A)** Hematoxylin and eosin images (original magnification, a1 x10, a2 x20) showing a thymoma composed of B3 histologic type (predominant, about 80%) and B2 (about 20%). B2 component thymoma showing mixture of small lymphocytes and large, polygonal epithelial cells, some of which are forming clusters of 3 or more epithelial cells (original magnification, a3 x20). B3 component thymoma showing cellular lobules comprised of large polygonal neoplastic cells and a few small lymphocytes (original magnification, a4 x20). (The slides were digitized with an Aperio AT2 scanner with 40x optics). **(B)** The immunohistochemical images staining for p40, p63, CK AE1/AE3, TdT, CD5, CD117, ki67 (original magnification, x10). (The slides were digitized with an Aperio AT2 scanner with 40x optics). **(C)** Hematoxylin and eosin image (original magnification, c1 x0.3, c2 x4, c3 x10, cd x20) showing extensive neoplastic infiltration composed of large poorly differentiated epithelial cells, round to oval-shaped with focal squamous aspects and some highly pleomorphic. (The slides were digitized with an Aperio AT2 scanner with 40x optics). **(D)** The tumor stains for cytokeratin AE1-AE3, Ki67, PD-L1, GLUT-1, p40, p53 and p63 proteins and it's negative for CD117/ c-Kit and CD5 (original magnification, 10x). (The slides were digitized with an Aperio AT2 scanner with 40x optics).

In October 2002, a patient in their 50s underwent radical thymectomy (R0) to remove a mediastinal mass. Histology showed B2/B3 stage III thymoma according to the Masaoka-Koga classification (**Figures 1A, B**). In November 2002, the patient was referred to the Regional Coordinating Center for Rare Tumors (CRCTR) of the Campania Region, in Italy. At the time of referral, the patient had an ECOG performance status of 0. Immunological screening revealed a diagnosis of GS.

**Figure 2:**
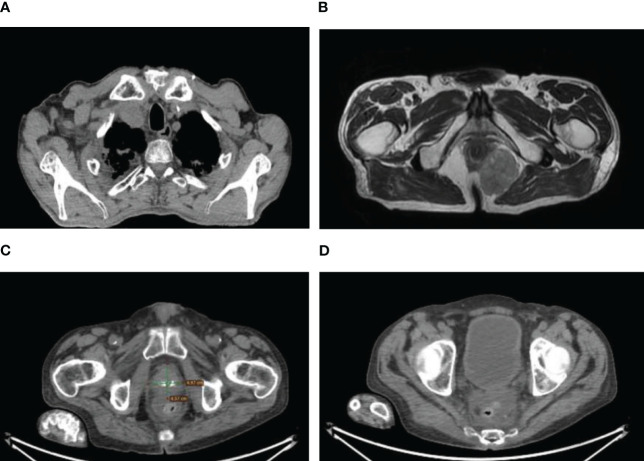
**(A)** Right supraclavicular lymphadenopathy. **(B)** Pararectal metastasis from thymoma at CT. **(C)** Prostate cancer. **(D)** Rectal cancer.

After thymectomy, the patient underwent four cycles of adjuvant chemotherapy consisting of carboplatin (AUC6 on day 1) and etoposide (100 mg/m2 on days 1–3). Subsequent surveillance did not show any signs of recurrence until August 2014. At that time, radiological evaluation, including fluorodeoxyglucose (FDG)-positron emission tomography (PET) and computed tomography (CT), revealed lymphadenopathy in the right supraclavicular region and a sizable expansive lesion (measuring 45x46x39 mm) in the anterior mediastinum, which was indicative of recurrence (**Figure 2A**). Fine needle cytology (FNC) of the supraclavicular node confirmed the recurrence of the thymoma with histopathological features consistent with those of the primary tumor.

The first-line treatment for metastatic disease was initiated with cisplatin (50 mg/m2 on day 1), doxorubicin (50 mg/m2 on day 1), and cyclophosphamide (500 mg/m2 on day 1). However, the treatment was halted prematurely due to an anaphylactic reaction during the first infusion. Consequently, the patient underwent six cycles of carboplatin (AUC 6) and paclitaxel (175 mg/m2) from November 2014 to January 2015, which resulted in a radiological partial response. In November 2015, subsequent progression prompted the administration of six cycles of cisplatin (100 mg/m2 on day 1) plus 5-fluorouracil (1000 mg/m2 on days 1–4), followed by radiation therapy to the residual mediastinal mass (50 Gy in 25 fractions with an additional boost of 10 Gy in 5 fractions on neoplasia), achieving stable disease.

From May 2015 to March 2017, the patient’s disease progressed, requiring several treatments. These included oral etoposide (6 cycles), cisplatin plus 5-fluorouracil and cetuximab (6 cycles), and SSA-LAR plus prednisone (4 months), which achieved stable disease with the best response. During follow-up in October 2016, a suspicious para-rectal lesion was detected (**Figure 2B**). Colonoscopy yielded negative results, leading to radical surgical excision (R0) in March 2017. Histopathological examination revealed extensive infiltration by a poorly differentiated carcinoma, which was likely of thymic origin, with focal squamous differentiation. Immunohistochemical analysis revealed positivity for pan cytokeratin (CKAE1/AE3), glucose transporter-1 (Glut-1), p63, p40, and p53 proteins, with Programmed Death-1 ligand (PD-L1) defined as combined positive score (CPS) ≥ 1%. CD5 and CD117/c-Kit were negative (**Figures 1C, D**). Immunohistochemistry for Glut-1 on pararectal lesions suggested the presence of a pararectal metastasis of a TET, representing a progression from thymoma to thymic carcinoma (6). Genetic analyses of the two neoplasias could not be performed, however, rarely thymic carcinomas were reported to arise from type B3 thymomas by molecular analysis. Moreover, by extensive clinical and radiological investigation, no other primary tumor was found in the patient (7).

After disease progression, the patient received sixth-line systemic platinum-based chemotherapy (carboplatin AUC 5 on day 1 and gemcitabine 1000 mg/m2 on days 1 and 8 every 21 days) from August 2017 to November 2017. However, lymph node progression occurred after five cycles, leading to the initiation of off-label treatment with the anti-programmed death 1 (PD-1) ICI nivolumab. The treatment was stopped after only two cycles because the patient developed heart failure and dyspnea. Diagnostic tests revealed a grade 4 myocarditis, which was complicated by atrial fibrillation with high ventricular response. The patient was treated with high-dose methylprednisolone and antiviral therapy in the infectious disease unit due to cytomegalovirus (CMV) reactivation. The patient achieved a radiological and clinical complete response nine months after stopping immunotherapy.

**Figure 3:**
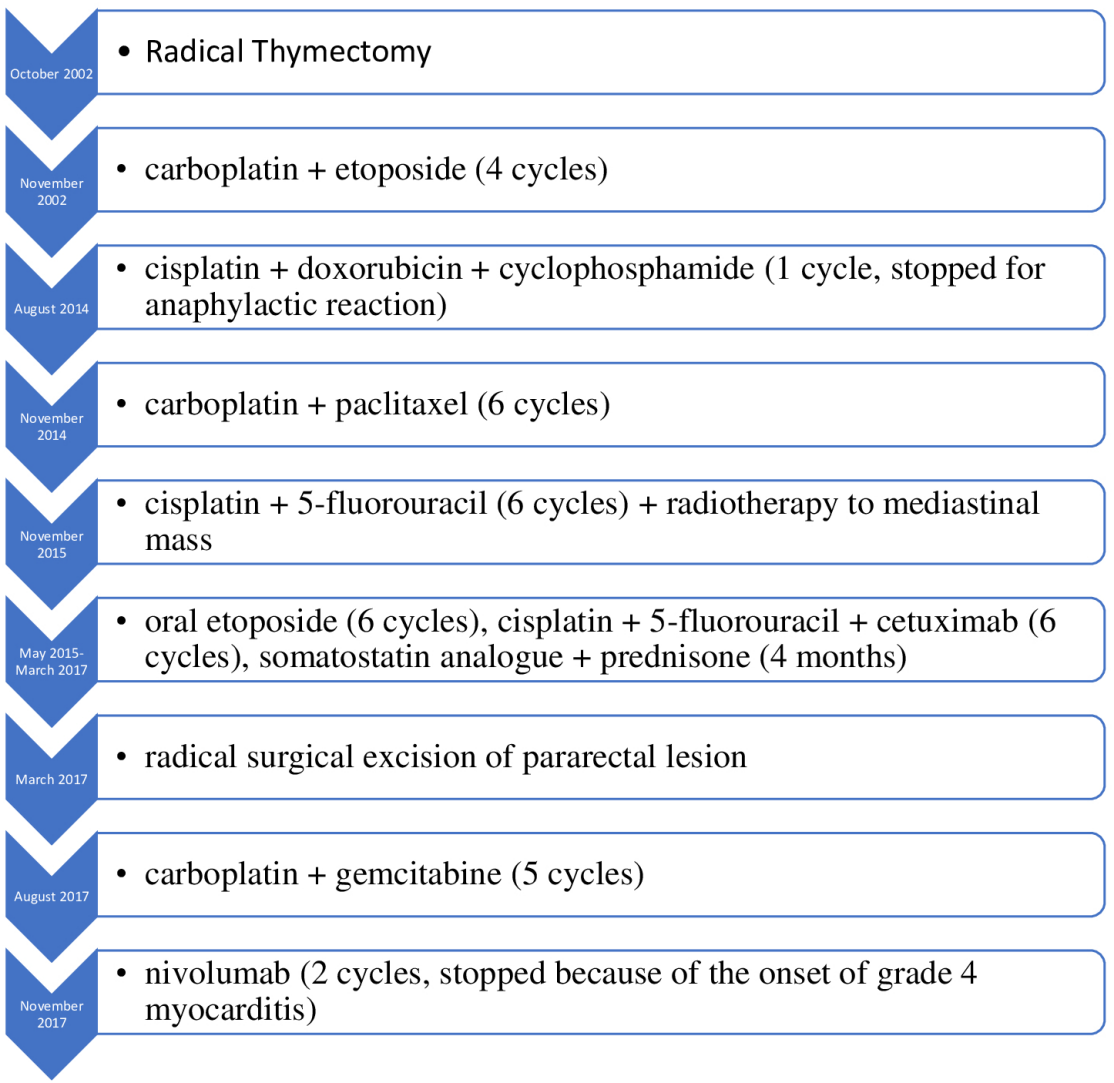
Timeline of treatments.

In May 2020, the patient was diagnosed with prostate cancer (Gleason score 9) and received radiation therapy followed by androgen deprivation therapy (**Figure 2C**). Timeline of treatments is summarized in **Figure 3**.

In October 2022, the patient was diagnosed with primary rectal adenocarcinoma and underwent radical surgery (**Figure 2D**). Lynch syndrome was excluded through mismatch repair (MMR) gene testing. The patient died in January 2024 due to heart failure, with no evidence of thymoma or rectal adenocarcinoma recurrence.

## Discussion

In this report, we described the extensive medical history of a patient with thymoma and GS who underwent locoregional treatment and multiple systemic treatment regimens, including an ICI.

Immunotherapy plays a potential role in the management of TETs due to the expression of PD-1 and programmed death–ligand 1 (PD-L1) on cancer cells. Several trials have investigated the impact of immunotherapy in patients with TETs, but few have reported complete responses. In a single-arm phase II trial conducted by Giaccone et al., 41 patients with advanced, refractory or recurrent thymic carcinoma were treated with pembrolizumab; these patients achieved mostly partial response (20%) and stable disease (53%), and only one patient achieved a complete response (3%) (8). Importantly, this complete response did not occur after only two cycles, as observed in our patient. This finding presents a significant challenge to elucidate: could previous radiotherapy potentially enhance the response to immunotherapy through an abscopal effect? This phenomenon could be explained by the modulation of the immune system’s response to cancer cells induced by prior radiation exposure, creating a more favorable environment for subsequent immunotherapy. Therefore, the combination of treatments with different functional mechanisms, such as immunotherapy and radiotherapy, can lead to more severe treatment-related adverse events (8).

Compared to other tumors, TETs are more frequently associated with immune-related adverse events (irAEs). The most commonly reported irAE is myasthenia gravis, which occurs in 24.5% of thymomas, while myocarditis is observed in 57% of thymomas and in 5% of TCs. These events may manifest with either asymptomatic clinical presentations or severe features (9).

Furthermore, our patient experienced a pararectal metastasis, an exceptionally rare site for recurrence, characterized by squamous and poorly differentiated neoplasia, likely of thymic origin. To the best of our knowledge, there are no reports in the literature documenting pararectal metastasis from thymic cancer. Indeed, dedifferentiation from thymoma to thymic carcinoma is a rare phenomenon. The expression pattern of Glut-1 differs between thymoma B3 and TC, exhibiting strong positivity in TCs but weak positivity in thymomas (10).

The observed shift toward an aggressive phenotype in our clinical case could justify the development of subdiaphragmatic metastasis as well as the remarkable response to immunotherapy.

Finally, our patient developed two additional metachronous tumors. However, the pathogenic mechanism underlying the association between thymoma and additional neoplasms remains unclear. Differences in histology within thymic cancer tissue, along with disruptions in T-cell development, may contribute to immune dysfunction, impairing immune surveillance and fostering uncontrolled cancer proliferation (11). Nevertheless, further studies are needed to investigate this association.

## Conclusions

TETs are complex clinical entities requiring multimodal therapeutic approaches. This is the first clinical case demonstrating a prolonged complete response in a thymoma treated with two cycles of anti-PD1 therapy with, as expected, a subsequent life-threatening immune-related adverse event successfully resolved in a center with high expertise in TET management.

Significantly, our patient exhibited a robust response to anti-PD1 therapy, resulting in prolonged survival despite severe toxicity. This emphasizes the crucial need for thorough scientific investigation to delineate innovative strategies for reducing both the risk and severity of immune-related adverse events in patients with TETs undergoing treatment with ICIs.

## Data availability statement

The raw data supporting the conclusions of this article will be made available by the authors, without undue reservation.

## Ethics statement

Written informed consent was obtained from the individual(s) for the publication of any potentially identifiable images or data included in this article.

## Author contributions

AL: Visualization, Validation, Writing – review & editing, Writing – original draft. EP: Visualization, Validation, Writing – review & editing, Writing – original draft. MO: Writing – review & editing, Visualization, Validation. AG: Writing – review & editing, Validation. AP: Writing – review & editing, Visualization, Validation. PDP: Writing – review & editing, Visualization, Validation. AS: Writing – review & editing, Visualization, Validation. MM: Writing – review & editing, Visualization, Validation. SV: Writing – review & editing, Visualization, Validation. RB: Writing – review & editing, Visualization, Validation, Supervision. GP: Writing – review & editing, Visualization, Validation, Supervision, Data curation, Conceptualization. MG: Writing – review & editing, Visualization, Validation, Supervision, Data curation.

## References

[1] Tartarone A Lerose R Lettini AR Tartarone M. Current treatment approaches for thymic epithelial tumors. Life. (2023) 13:1170. doi: 10.3390/life13051170 37240815 PMC10222654

[2] Marx A Chan JKC Chalabreysse L Dacic S Detterbeck F French CA. The 2021 WHO classification of tumors of the thymus and mediastinum: what is new in thymic epithelial, germ cell, and mesenchymal tumors? J Thorac Oncol Off Publ Int Assoc Study Lung Cancer. (2022) 17:200–13. doi: 10.1016/j.jtho.2021.10.010 34695605

[3] Imbimbo M Ottaviano M Vitali M Fabbri A Leuzzi G Fiore M. Best practices for the management of thymic epithelial tumors: A position paper by the Italian collaborative group for ThYmic MalignanciEs (TYME). Cancer Treat Rev. (2018) 71:76–87. doi: 10.1016/j.ctrv.2018.10.001 30366202

[4] Malfitano AM D’Esposito V De Placido P Tortora M Ottaviano M Pietroluongo E. Immunological signature of patients with thymic epithelial tumors and Good syndrome. Front Immunol. (2022) 13:908453. doi: 10.3389/fimmu.2022.908453 36059463 PMC9434000

[5] Pietroluongo E De Placido P Tortora M Martinelli C Viggiano A Saponaro MR. Impaired seroconversion after severe acute respiratory syndrome coronavirus 2 mRNA vaccine in patients with thymic epithelial tumors. J Thorac Oncol Off Publ Int Assoc Study Lung Cancer. (2023) 18:1399–407. doi: 10.1016/j.jtho.2023.06.015 PMC10303630 37390981

[6] Sutedja EK Rizqandaru T Ruchiatan K Sutedja E. Cutaneous metastases from thymic carcinoma primary tumor: A rare case. Int Med Case Rep J. (2022) 15:293–8. doi: 10.2147/IMCRJ.S369726 PMC9207123 35734095

[7] Massoth LR Hung YP Dias-Santagata D Onozato M Shah N Severson E. Pan-cancer landscape analysis reveals recurrent KMT2A-MAML2 gene fusion in aggressive histologic subtypes of thymoma. JCO Precis Oncol. (2020) 4:PO.19.00288. doi: 10.1200/PO.19.00288 32923872 PMC7446345

[8] Ao Y-Q Gao J Wang S Jiang J-H Deng J Wang H-K. Immunotherapy of thymic epithelial tumors: molecular understandings and clinical perspectives. Mol Cancer. (2023) 22:70. doi: 10.1186/s12943-023-01772-4 37055838 PMC10099901

[9] Kelly RJ Petrini I Rajan A Wang Y Giaccone G. Thymic Malignancies: from clinical management to targeted therapies. J Clin Oncol. (2011) 29:4820–7. doi: 10.1200/JCO.2011.36.0487 PMC3675690 22105817

[10] Du MJ Shen Q Yin H Rao Q Zhou MX. Diagnostic roles of MUC1 and GLUT1 in differentiating thymic carcinoma from type B3 thymoma. Pathol Res Pract. (2016) 212:1048–51. doi: 10.1016/j.prp.2016.09.005 27688088

[11] Welsh JS Thurman SA Howard SP. Thymoma and multiple Malignancies: A case of five synchronous neoplasms and literature review. Clin Med Res. (2003) 1:227–32. doi: 10.3121/cmr.1.3.227 PMC1069048 15931312

